# Self-sustained oscillations and global climate changes

**DOI:** 10.1038/s41598-020-68052-9

**Published:** 2020-07-08

**Authors:** Luis G. Arnaut, Santiago Ibáñez

**Affiliations:** 1 Chemistry Department, University of Coimbra, Coimbra, Portugal; 2 Departamento de Matemáticas, Universidad de Oviedo, Oviedo, Spain

**Keywords:** Climate change, Chemistry, Mathematics and computing

## Abstract

The periodic changes of atmospheric CO_2_ and temperature over the last 5 Myr reveal three features that challenge current climate research, namely: (i) the mid-Pleistocene transition of dominant 41-kyr cycles to dominant 100-kyr cycles, (ii) the absence of a strong precession signal of approximately 20 kyr, and (iii) the cooling through the middle and late Holocene. These features are not directly addressable by Earth’s orbital changes described by Milankovitch. Here we show that a closed photochemical system exposed to a constant illumination source can sustain oscillations. In this simple conceptual model, the oscillations are intrinsic to the system and occur even in the absence of periodic radiative forcing. With proper adaptations to the Earth system, this oscillator explains the main features of past climate dynamics. Our model places photosynthesis and the carbon cycle as key drivers of climate change. We use this model to predict the relaxation of a 1,000 PgC pulse of CO_2_. The removal of 50% of this CO_2_ will require one century, and will lead to a warmer and wetter future. However, more pronounced glaciation cycles emerge on the millennial timescale.

## Introduction

Current atmospheric CO_2_ levels are the highest of the last 2 million years (Myr)^1^. The identification of the causes and prediction of the consequences of such high CO_2_ levels triggered intense scrutiny of drivers of past climate changes^2–6^. The same drivers should be present today and, together with new anthropogenic drivers, need to be considered in models aiming at the prediction of climate changes. The most prominent features of climate change over the last 5 Myr are the global cooling by 2–3 °C between 5.3 and 0.8 Myr ago, the change from a dominant periodicity of 40 kyr between 5 and 3.5 Myr ago to a dominant periodicity of 100 kyr in the last 800 kyr, and the occurrence of glaciations cycles at both poles in the last 800 kyr^1,7–10^. The power spectra of temperature changes captured by Antarctic ice records over the last 420 to 720 kyr confirms the dominance of 100-kyr period followed by minor 40-kyr and ca. 23-kyr periods^11,12^. The change in the dominant periodicity from approximately 40 kyr to approximately 100 kyr is known as the middle Pleistocene transition (MPT).

**Figure 1:**
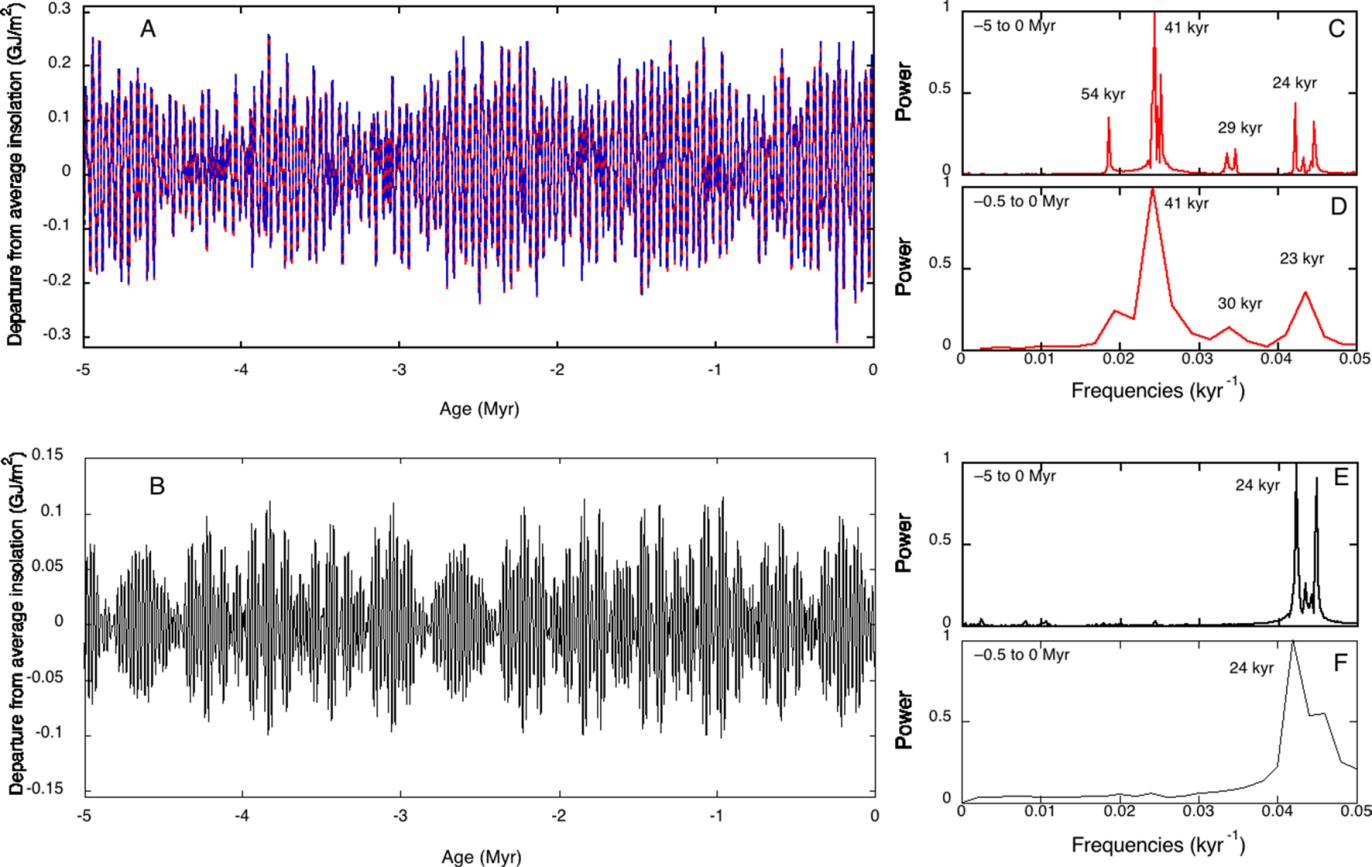
(**A**) Changes in summer energies at 65°N above the insolation threshold of 275 W/m^2^ relative to average (ca. 5 GJ/cm^2^ ^17^, centered in zero, red line), and corresponding Fourier series (blue dashed line). (**B**) Changes in solar energy received at 25°N in the month of March relative to average (ca. 1 GJ/cm^2^, centered in zero)^18,19^. Corresponding frequency distributions calculated for the time interval from – 5 Myr to present (**C**,**E**), or for the last 500 kyr (**D**,**F**).

Explanations of past climate changes, namely of glaciation cycles, continue to favor orbital forcing^13,14^ due to Milankovitch cycles^15^, possibly amplified first by greenhouse gases and then by deglaciation and ice-albedo feedback. Milankovitch cycles relate cyclic variations in insolation to three main orbital parameters: eccentricity (Earth’s orbit changes with periods of 96 and 125 kyr), obliquity (changes in the tilt of the Earth’s axis of rotation with period of 41 kyr) and precession (precession of the equinoxes and movement of the perihelion, with periodicities of 23 and 19 kyr). Precession dominates insolation mainly in the equatorial regions, with the contribution of obliquity reinforced at the solstices and at high latitudes^16^, while eccentricity has only a very weak effect on insolation. Figure 1 shows changes in summer energy at 65°N^17^ or in March at 25°N^18,19^ over the last 5 Myr with respect to their average. Their frequency distributions reveal the irrelevance of eccentricity. Climate changes driven by orbital forcing in the last 800 kyr would require nonlinear effects to amplify insolation changes due to eccentricity that are not present in obliquity or in precession. This anomaly of the astronomical forcing hypothesis is widely recognized^17,20–22^.

Figure 1 also shows that the oscillations of summer energy did not systematically depart from the 5 GJ/cm^2^ average in the last 5 Myr, but eight glacial-interglacial cycles occurred over the last 800 kyr. Attempts to explain these intriguing observations have followed two strategies^23^. One approach argues that the 100 kyr periods are in fact cycles of approximately 80 kyr or approximately 120 kyr quantized in multiples of the basic obliquity cycle^24^, and relies on frequency-locking of obliquity and/or precession to an external forcing to allow for skipping some of the obliquity cycles^6^. The other approach^3^ associates MPT with an oscillator with a limit-cycle of 100 kyr which, for example, with a linear decrease in atmospheric CO_2_ reaches an abrupt bifurcation between the linear response to obliquity forcing and the oscillator regime^25^. However, three major issues remain unresolved: why did ice sheets start to develop over the Northern Hemisphere approximately 3.3–2.7 Myr ago? Why did climate cycles evolve from 40- to 100-kyr periodicities ca. 1 Myr ago? What is the origin of the dominant 100-kyr frequency of the last 5 glacial cycles? Abandoning the hypothesis of orbital forcing raises an even more fundamental question: how can a closed system such as Earth trigger and sustain oscillations on the millennia time scale? The answer to this question should point to a physical mechanism capable of sustained oscillations in a closed system such as Earth.

According to the second law of thermodynamics, in an *isolated system*, exchanging neither energy nor matter with the outside world, entropy increases monotonically to its maximum at equilibrium. An *open system* can exhibit persistent oscillations of some of its components because there is a constant influx of another component. The first efforts to find open systems with undamped oscillations appeared in chemical sciences and were authored by Lotka^26^, but it was the explanation of Volterra for predator–prey population relations^27^ that popularized undamped oscillations. Prigogine demonstrated the possibility of having chemical oscillations of intermediates of an overall reaction A + B → C + D^28^. The mechanism proposed, named *Brusselator*^29^, is the prototypical model of an autocatalytic chemical reaction providing oscillations and even chaos when two *Brusselators* are coupled^30^. The first experimental observation of oscillations was the Belousov-Zhabotinsky (BZ) reaction^31^, that undergoes approximately 100 oscillations before achieving the final equilibrium state. Field and Noyes proposed a general kinetic scheme, named *Oregonator*, to explain such oscillations and showed that sustained oscillations required some chemical components to remain constant^32^, i.e., sustained oscillations required influx of material. This led to the believe that a *closed system* at constant temperature and pressure should attain equilibrium^33^. Sustained oscillations were only anticipated for open systems described by a set of non-linear chemical reactions with autocatalysis and feedback^34^.

Earth is a (nearly) closed system receiving an influx of energy from the Sun. The oscillators mentioned above cannot represent an internal Earth system mechanism with glaciation cycles. However, a closed chemical reaction system where a net product of other steps is photochemically transformed back in the reactant may have sustained oscillations^35^. Photosynthesis can maintain the state of disequilibrium on Earth, but storage and release of free energy alone are insufficient to drive oscillations. Persistent oscillations also require autocatalysis, where one component X is a catalyst of its own production, D + X → Z + 2X.

We present a simple conceptual model for intrinsic oscillations in a closed system under constant illumination, such as the Earth, that accounts for the major trends of temperature, CO_2_ and biomass cycles over the last 5 million years. We design the simplest oscillator for a closed system that can sustain periodic changes of its composition under constant illumination, and then add the minimum complexity required to represent the Earth system. The model only employs two adjustable parameters, which are fitted to historic CO_2_ cycles, but incorporates a mechanism that triggers oscillations. This trigger is associated with a change in the global efficiency of photosynthesis in the Pliocene. The oscillations are enabled by an autocatalytic step. The dominant periodicity of 100 kyr arises naturally from the timescales of CO_2_ land uptake, ocean invasion, reaction with calcium carbonate and silicate weathering, once the efficiency of photosynthesis increased. We use this model to predict long-term climate consequences of a large CO_2_ pulse.

## Results

### Oscillations in a closed system under illumination

Dynamical systems are often described by a set of ordinary differential equations (ODEs) that contain parameters in addition to variables. In some systems, similar sets of parameter values lead to dynamic behaviors of qualitatively different nature. For example, one set of parameters may lead to a stable equilibrium point—an attractor—whereas the other set leads to an asymptotically stable periodic solution—a limit cycle. Such models are said to contain bifurcation points. The case given above, where an attractor becomes a limit cycle as a parameter is varied, is named a supercritical Hopf bifurcation. When such a bifurcation is present, the system may exhibit exponentially damped oscillations leading to equilibrium or to limit cycle oscillations^36^. These limit cycles are periodic orbits when represented as trajectories in the phase space of the variables. Chemical oscillations arise from Hopf bifurcations in the *Oregonator*^32^, in the oscillations of glycolysis^37^ and in the *Brusselator*^29^.

**Figure 2:**
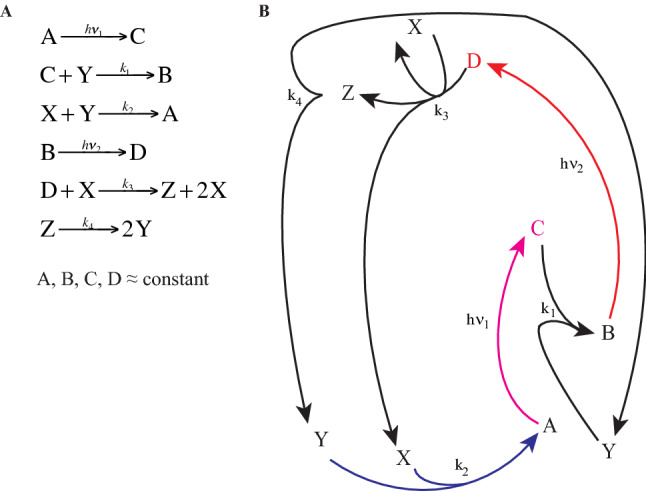
The *Coimbrator*. Representation of the reactions taking place in the closed system, where C is obtained from A by electronic excitation with *hν*_1_, and D is obtained from B by electronic excitation with *hν*_2_. The photochemical steps are shown in red and raise the free energy of the system. Under conditions where A, B, C and D are approximately constant, the changes in the concentrations of the composition variables *X*, *Y*, and *Z* are determined by the reactions ${\text{C}} + {\text{Y}}\mathop{\longrightarrow}\limits^{{k_{1} }}{\text{B}},{\text{X}} + {\text{Y}}\mathop{\longrightarrow}\limits^{{k_{2} }}A,{\text{D}} + {\text{X}}\mathop{\longrightarrow}\limits^{{k_{3} }}{\text{Z}} + 2{\text{X}}\,{\text{and}}\,{\text{Z}}\mathop{\longrightarrow}\limits^{{k_{4} }}2{\text{Y}}$.

Following the tradition of naming oscillators based on their geographical origin, we name *Coimbrator* the set of ODEs represented in Fig. 2. They enable “self-oscillations” of composition variables as a parameter crosses a critical value. Figure 2 emphasizes that the *Coimbrator* is a closed system with two photochemical steps. The equations are at most bimolecular, contain three composition variables (X, Y, Z) and include an autocatalytic reaction, which are the minimal sufficient conditions for realistic oscillatory behavior^38^.

This closed system can be simplified when the concentrations of dyes *A* and *B* are high compared with the photon flux, and can be considered constant. Moreover, if the photon fluxes with energies *hν*_1_ and *hν*_2_ are also approximately constant, the concentrations of *C* and *D* will remain approximately constant (steady-state). Hence, for large *A* and *B* and constant illumination, *A*, *B*, *C* and *D* can be treated as constants and the system can be decoupled from the variations of *X*, *Y* and *Z*. “Methods” section shows how to express these species in dimensionless variables (*x*, *y*, *z*) to obtain the following ODEs

1 $$\begin{aligned} & \dot{x} = \alpha x - xy \\ & \dot{y} = - y - xy + 2\beta z \\ & \dot{z} = \alpha x - \beta z \\ \end{aligned}$$

where the dot notation denotes the derivative with respect to dimensionless time; *α* = (*k*_3_D)/(*k*_1_C) and *β* = *k*_4_/(*k*_1_C) are parameters. We define 1/(*k*_1_*C*) as the characteristic time.

**Figure 3:**
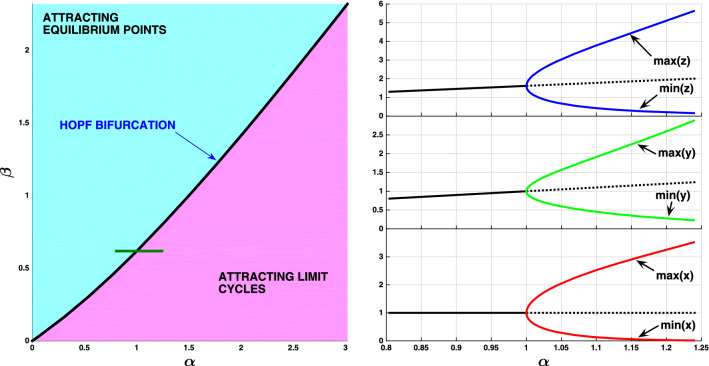
Bifurcation diagram of the Coimbrator. Left panel: the Hopf bifurcation is supercritical and the oscillations arise for parameter values to the bottom-right side of the bifurcation curve. Right panel: the onset of the cycle as *α* increases along the green segment (*β* = 0.6180) depicted in the left panel (*z* = blue, *y* = green, *x* = red).

The equilibrium (fixed) points of this three-dimensional system are obtained solving $\dot{x}$ = 0, $\dot{y}$ = 0 and $\dot{z}$ = 0 simultaneously. They are *O* = (0,0,0) and *P* = (1,* α*,* α/β*). When *β* < [*α*–2 + √(*α* + 4)]/2 the point *P* becomes a repelling equilibrium point and a periodic orbit appears. “Methods” section presents the treatment of the bifurcation. The Hopf bifurcation is supercritical in the whole region depicted in Fig. 3. Attracting limit cycles emerge when crossing the bifurcation curve with $\beta$ decreasing or *α* increasing. Figure 3 illustrates the bifurcation selecting a segment in the parameter space *β* = 0.6180 and *α* ∈ [0.80, 1.25].

**Figure 4:**
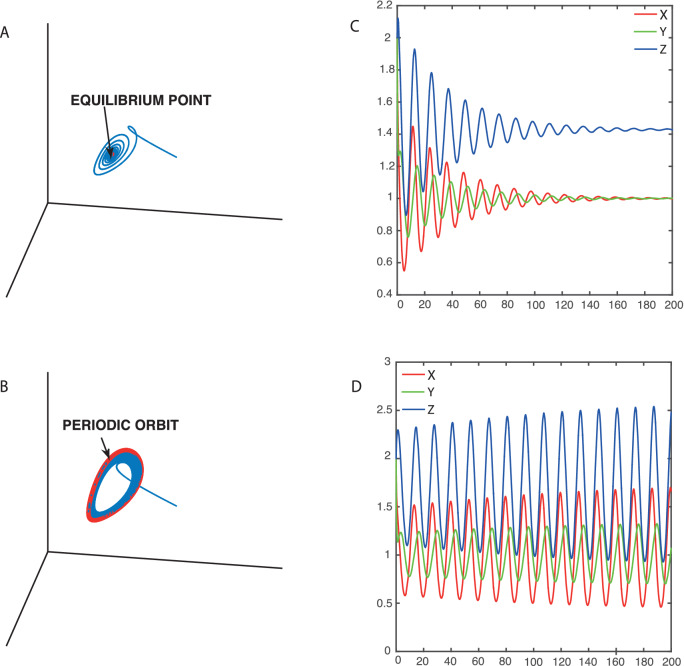
Phase space plots obtained by solving numerically the differential equations of the Coimbrator. (**A**) Dynamics with *α* = 1 and *β* = 0.7 make the system evolve to equilibrium, and (**C**) corresponding damped oscillations of the *xyz* variables. (**B**) Dynamics with *α* = 1 and *β* = 0.6 lead to a periodic orbit and (**D**) corresponding sustained oscillations of the *xyz* variables.

Figure 4 presents phase space trajectories to illustrate the crossing of the supercritical Hopf bifurcation. The numerical simulations in the upper panel illustrate a trajectory for certain values of the parameters (e.g.,* α* = 1 and *β* = 0.7) that is attracted to an equilibrium point. This is true independently of the initial values of the variables (*x*, *y*, *z*). The trajectories in the lower panel calculated with another set of parameters (e.g., *α* = 1 and *β* = 0.6) go to an asymptotically stable periodic orbit. A Hopf bifurcation occurs for *β* = 0.618 when *α* = 1. Figure 4 also represents the values of the variables as a function of time. For *α* = 1 and *β* = 0.7 we see exponentially damped oscillations of the variables leading to a final stable value. This closed system sustains oscillations of the variables for *α* = 1 and *β* < 0.618. The *Coimbrator* leads to oscillations when *β* crosses a critical value.

### The Earth system as an oscillator

The *Coimbrator* shows that the internal Earth system mechanism that drives climate changes can be an oscillator with parameters that in the Pleistocene crossed those of a supercritical Hopf bifurcation and triggered glacial cycles. We explore this hypothesis incorporating the most essential elements of the Earth system in the oscillator.

Photosynthesis in plants involves two photons of different energies to produce a high-energy intermediate,

2 $$2\left( {{\text{CO}}_{2} + 2{\text{H}}_{2} {\text{O}} + {\text{photon}} \to {\text{CH}}_{{2}} {\text{O}} + {\text{H}}_{{2}} {\text{O}} + {\text{O}}_{{2}} } \right)$$

This overall reaction of oxygenic photosynthesis represents the buildup of biomass. Hence, we identify CH_2_O with *Z*. It represents both live biomass and dead organic carbon. Its consumption is the oxidation reaction

3 $$2\left( {{\text{CH}}_{2} {\text{O}} + {\text{O}}_{{2}} \mathop{\longrightarrow}\limits^{{k_{4} }}{\text{CO}}_{{2}} + {\text{H}}_{{2}} {\text{O}}} \right)$$

The amount of oxygen in the atmosphere decreased by less than 1% in the last 5 million years and remained at least one order of magnitude higher than that of H_2_O or CO_2_. Assuming that O_2_ is approximately constant and can be assimilated in *k*_4_, the autocatalysis reaction becomes

4 $${\text{D}} + {\text{H}}_{2} {\text{O}}\mathop{\longrightarrow}\limits^{{k_{3} }}{\text{CH}}_{2} {\text{O}} + {\text{2H}}_{2} {\text{O}}$$

where *D* comprises elements from the photosynthetic systems and carbon fixation cycle. A, B and C are also part of this carbon fixation cycle. This leads to the assignment of *X* as water vapor and, consequently, *Y* = CO_2_. This approach emphasizes the analogy with the *Coimbrator* and helps to identify both the photochemical reaction that drives the system and the autocatalysis required for oscillations. These aspects and the presence of a quadratic term, make the representation of the oscillator as a compartmental model that exchanges substances between physical spaces (e.g., atmosphere, sea, biosphere) less insightful than the representation of Fig. 2.

The main control of water vapor in the atmosphere is the vapor–liquid (*X* ↔ *X*_0_) equilibrium. We add the (*X* ↔ *X*_0_) equilibrium to the oscillator to account for the saturation of water vapor pressure in moist air. Similarly, the CO_2_ solubility (*Y* ↔ *Y*_0_) equilibrium is included to the oscillator, together with the reactions of CO_2_ dissolved in water, CO_2(aq)_. “Methods” section show how choices of physically motivated parameters constrain changes in *X* and *Y*. Adding these equilibria to the oscillator changes its nature. We name the new oscillator *Glaciator*.

**Figure 5:**
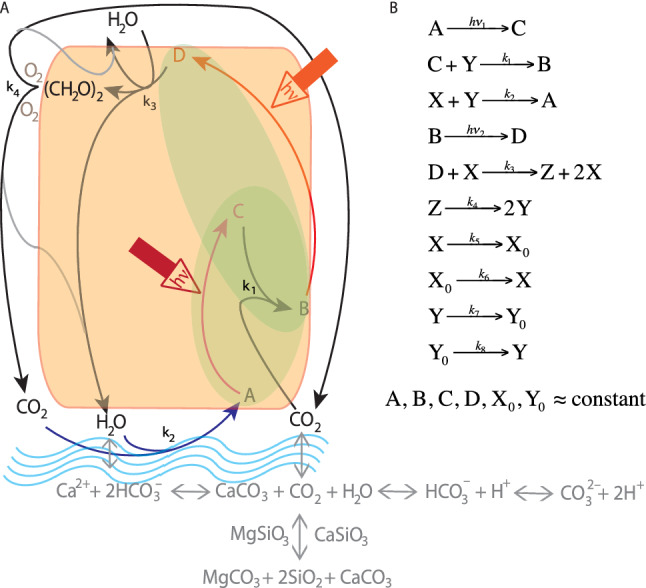
The *Glaciator*. Representation of the reactions taking place in the closed system emphasizing the analogy between photochemical reactions and photosynthesis, and their generation of a high-energy intermediate (CH_2_O)_2_. The green shade covers the photosynthetic steps and the orange shade covers the steps in the biomass. Under conditions where A, B, C and D are approximately constant, the changes in the concentrations of the composition variable *X* = H_2_O, *Y* = CO_2_ and *Z* = CH_2_O are determined by the reactions of the *Coimbrator* and, additionally, by ${\text{X}}\mathop{\longrightarrow}\limits^{{k_{5} }}{\text{X}}_{0} ,{\text{X}}_{0} \mathop{\longrightarrow}\limits^{{k_{6} }}{\text{X}},{\text{Y}}\mathop{\longrightarrow}\limits^{{k_{7} }}{\text{Y}}_{0} \,{\text{and}}\,{\text{Y}}_{0} \mathop{\longrightarrow}\limits^{{k_{8} }}{\text{Y}}$.

Figure 5 illustrates how the *Glaciator* incorporates the basic features of the Earth system. “Methods” section shows how to formulate the *Glaciator* in terms of dimensionless variables. Assuming that *X*_o_ and *Y*_0_ are constant, the new set of ODEs involves four parameters (*ε*, *σ*, *ω*, *ρ*)

5 $$\begin{array}{*{20}l} {{\text{Water vapor }}\left( {x = {\text{H}}_{2} {\text{O}}} \right)} \hfill & {\dot{x} = \varepsilon + \left( {\alpha - \sigma } \right)x - xy} \hfill \\ {{\text{Carbon dioxide }}\left( {y = {\text{CO}}_{2} } \right)} \hfill & {\dot{y} = \omega - \left( {1 + \rho } \right)y - xy + 2\beta z} \hfill \\ {{\text{Organic carbon }}\left( {z = {\text{CH}}_{{2}} {\text{O}}} \right)} \hfill & {\dot{z} = \alpha x - \beta z} \hfill \\ \end{array}$$

where *ε* = (*k*_6_*x*_0_)/(*k*_1_*C*), *σ* = *k*_5_/(*k*_1_*C*), *ω* = (*k*_8_*y*_0_)/(*k*_1_*C*) and *ρ* = *k*_7_/(*k*_1_*C*), in addition to *α* = (*k*_3_D)/(*k*_1_C) and *β* = *k*_4_/(*k*_1_C). The rates of the processes represented by *k*_3_D and *k*_4_ depend on the biomass, which is not constant, and the corresponding parameters are better treated as time dependent, *α*(*t*) and *β*(*t*). We recall that in the *Coimbrator* we assumed a constant irradiation source, and consequently constant *α* and *β*, but this is no longer valid when we account for the insolation changes of Fig. 1 in the *Glaciator*. The rate constants *k*_5_ and *k*_6_ for the (*X* ↔ *X*_0_) equilibrium depend on the temperature and, consequently, *ε* and *σ* should also be time dependent. For simplicity, that is not considered in this study.

Figure 5 represents the dissolution of carbonates explicitly for CaCO_3_ and MgCO_3_. Identically, CO_2_ reactions with calcium- or magnesium-containing silicate minerals (CaSiO_3_ or MgSiO_3_) are equally present. The overall reactions

6a $${\text{CO}}_{{2}} + {\text{ CaSiO}}_{{3}} \leftarrow \to {\text{CaCO}}_{{3}} + {\text{ SiO}}_{{2}}$$

6b $${\text{CO}}_{{2}} + {\text{ MgSiO}}_{{3}} \leftarrow \to {\text{MgCO}}_{{3}} + {\text{ SiO}}_{{2}}$$

represent from left-to-right Ca-Mg silicate weathering plus sedimentation of marine carbonates. From right-to-left these reactions represent thermal decomposition of carbonates at depth resulting in degassing of CO_2_ to the surface^39^.

### Timescales of climate changes

We show in “Methods” section that meaningful values of the parameters in Eq. (5) require *ε* ≈ 33*ω* and *σ* ≈ 3.3*ρ*. The independent parameters *α*(*t*), *β*(*t*), *ω* and *σ* are defined as ratios of rate constants (or time constants). This dependence on the rates places the timescales of the processes in Fig. 5 under careful scrutiny.

The major processes for CO_2_ removal from the atmosphere are those of land uptake (timescale 1–10^2^ yr), ocean invasion (10–10^3^ yr), reaction with calcium carbonate (10^3^–10^4^ yr) and silicate weathering (10^4^–10^6^ yr)^40^. In the *Glaciator*, CO_2_ removal is represented by

7 $$\begin{aligned} & {\text{C}} + {\text{CO}}_{2} \mathop{\longrightarrow}\limits^{{k_{1} }}B \\ & {\text{H}}_{2} {\text{O}} + {\text{CO}}_{2} \mathop{\longrightarrow}\limits^{{k_{2} }}{\text{A}} \\ & {\text{CO}}_{2} \mathop{\longrightarrow}\limits^{{k_{7} }}{\text{CO}}_{2} ({\text{aq}}) \\ \end{aligned}$$

We associate the rate of land uptake with

8 $$\frac{{{\text{d}}A}}{{{\text{d}}t}} = k_{2} p_{H2O} p_{CO2}$$

Considering that globally water vapor pressure is much larger than CO_2_ pressure, *p*_H2O_ ≫ *p*_CO2_, it is reasonable to incorporate the water vapor pressure in the rate constant and relate 1/(*k*_2_*p*_H2O_) to the timescale of CO_2_ removal by land uptake, 130 yr^41^. This, together with present day *p*_H2O_ ≈ 11 matm, discussed in “Methods” section, allow us to calculate *k*_2_ ≈ 0.7 atm^–1^ yr^–1^. We associate the timescale of silicate weathering with 1/*k*_7_ and make 1/*k*_7_ ≈ 100 kyr. This is also the residence time of inorganic carbon in the ocean^39^. The value of 1/(*k*_1_*C*), defined as the characteristic time, must be related to the timescales of ocean invasion and reaction with calcium carbonate. Admittedly, this step of the *Glaciator* could be divided in two steps, because at least these two physical processes are present. This would increase the complexity and the number of parameters in the calculations. We favored simplicity and use 1/(*k*_1_*C*) to describe the two processes and represent the mean atmospheric lifetime of CO_2_. The mean atmospheric lifetime of CO_2_ after a large emission pulse is 12–14 kyr^42^. Hence, we make 1/(*k*_1_*C*) ≈ 12 kyr, which is in the upper limit of the geometric mean of ocean invasion and calcium carbonate reaction timescales.

These timescales determine the characteristic concentration (*k*_1_*C*)/*k*_2_ = 0.12 matm, and allow for the conversion of the dimensionless variables (*x*, *y*, *z*) to absolute values of H_2_O, CO_2_ and biomass. The value of the parameter *ρ* is given by its definition, *ρ* = *k*_7_/(*k*_1_*C*), and using the timescales discussed above we obtain *ρ* = 0.11. This restricts the number of adjustable parameters to three: *α*(*t*), *β*(*t*) and *ω*. The parameter *ω* relates the timescale of CO_2_ release from the surface ocean to the atmosphere with its mean atmospheric lifetime. We employed *ω* = 5 but the calculations are not very sensitive to this value. The values of the timescales employed in this work are presented in Table 1. They lead to a peak in atmospheric CO_2_ in the Holocene before the onset of anthropogenic perturbations^43^.

**Table 1:** Parameters employed in the simulations with the *Glaciator*, including the methods used in their estimates.

Parameter	Value	Source
1 / (*k*_2_ *p*_H2O_)	130 yr	Timescale of land uptake
1 / (*k*_1_ *C*)	12,020 yr	Timescales of ocean invasion and reaction with CaCO_3_
1 / *k*_7_	108,420 yr	Timescale of silicate weathering
*ρ*	0.1109	*k*_7_/(*k*_1_ *C*)
*ω*	5	Fitted to historic CO_2_ cycles
*ε*	165	*ε* = 33*ω* from the ratio of H_2_O evaporation and CO_2_ desorption fluxes
*σ*	0.3659	*σ* = 3.3*ρ *from *ε* = 33*ω* and *p*_H2O_ ≈ 10*p*_CO2_
*α*(C4 plants)	1	Maximum efficiency of photosynthesis
*α*(C3 plants)	0.649	Radiation efficiency increased by 50% from C3 to C4 plants
*β*_0_	0.46	Fitted to historic CO_2_ cycles

**Figure 6:**
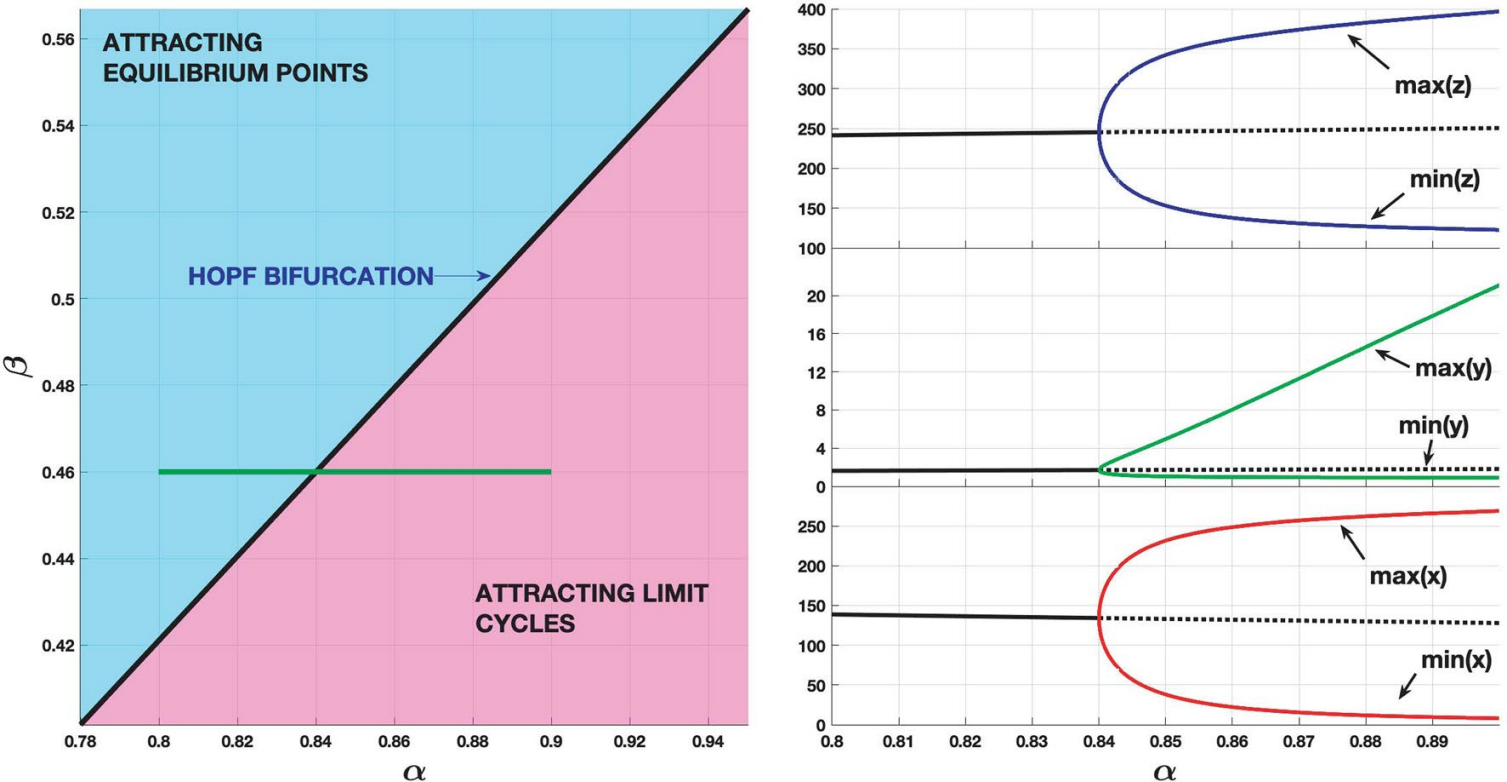
Bifurcation diagram of the *Glaciator*. Left panel: the Hopf bifurcation is supercritical and the oscillations arise for parameter values to the bottom-right side of the bifurcation curve. Right panel: the onset of the cycle as *α* increases along the green segment (*β* = 0.46) depicted in the left panel (*z* = blue, *y* = green, *x* = red).

“Methods” section present a study of the Hopf bifurcation in the *Glaciator*. For the fixed values *ω* = 5, *ρ* = 0.11, *ε* = 33*ω *and* σ* = 3.3*ρ*, the Hopf bifurcation is supercritical for the values of *α* and *β *shown in Fig. 6. This figure shows the crossing of the bifurcation curve when *β* = 0.46 and *α* increases in the segment *α* $\in$ [0.8,0.9]. The *Glaciator* may trigger sustained oscillations when *β* decreases or *α* increases from a set of parameters corresponding to a stable equilibrium.

### Photosynthetic forcing

Given the definition of *α* and the processes in Fig. 5, *α* can be regarded as the ratio between the rate of formation of the biomass and the rate of incorporation of CO_2_. Hence, *α* is a measure of the efficiency of photosynthesis and should not exceed unity.

There are two major types of photosynthesis, C3 and C4 photosynthesis, named after the number of carbon atoms of the first compound in which CO_2_ is incorporated. C3 plants (e.g., trees, wheat, rice, soybean) were spread in ancestral atmospheres, characterized by elevated CO_2_ and low O_2_ levels, whereas C4 plants (e.g., corn, sugarcane, many grasses) became dominant at mid-to-low latitudes in the last 5 Myr^44^, and now represent ¼ of the primary productivity on the planet^45^. C4 plants are able to concentrate CO_2_ and minimize photorespiration. This contributes to a specific activity (i.e., mol CO_2_ fixed per mass of enzyme per unit time) of C4 plants that can be twice as large as that of C3 plants^46^, and yields 50% greater efficiencies in radiation use^47^. Given the limit of *α*, we make *α*_C3_ ≈ 0.65 for C3 plants and, because the efficiency of C4 plants is 50% higher than that of C3 plants, we make *α*_C4_ ≈ 1. “Methods” section describe how to obtain *α*(*t*) from the changes in populations C3 and C4 plants. Using *β* = 0.46 and an increase in *α* from 0.84 to 0.925 over the last 10 Myr ago, we calculate that the Hopf bifurcation was crossed ca. 5 Myr ago and triggered a trajectory towards the limit cycle oscillation.

The value of *β* depends on production of (CH_2_O). Higher numbers of photons, larger areas covered by biomass and hydrological cycles driven by latitudinal insolation gradients should lead to temporary increases in *β*. Inter- or intra-hemispheric insolation gradients show the same features as the 65°N insolation curve, namely the dominant 41 kyr period that is the signature of obliquity^48–50^. We incorporate photosynthetic-orbital forcing in the *Glaciator* making *β*(*t*) = *β*_0_[1 + 0.09*F*(*t*)], where the zero of *F*(*t*) is the average summer energy of the Northern Hemisphere over the last 5 Myr (ca. 5 GJ/m^2^)^17^, and *F*(*t*) is normalized. The scaling factor 0.09 accounts for relative energy changes. A larger scaling factor could be used to account for the other mechanisms contributing to changes in *β*. Reasonably higher values did not change the nature of our results. This also means that choice of the insolation function is not critical for the results. The parameter *β*_0_ = 0.46 was selected to reproduce climate changes over the last 5 Myr. Its value implies that respiration, burning or other processes associated with the consumption of the biomass are in the same timescale as the incorporation of CO_2_ in the biomass.

**Figure 7:**
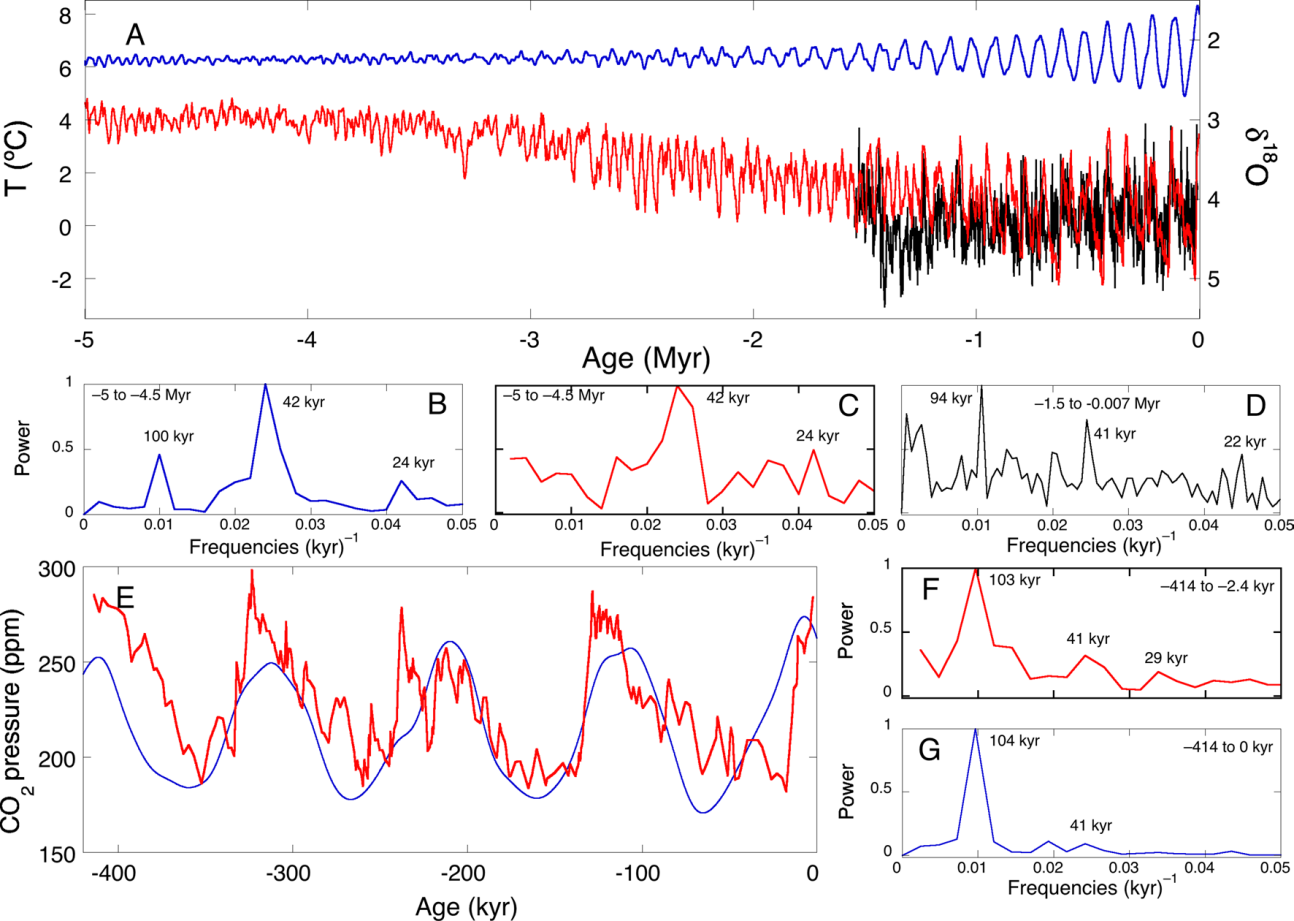
(**A**) Benthic δ^18^O records of the LR04 stack (red)^7^ matched to temperatures derived from Mg/Ca ratios of benthic foraminifera (black)^9^ and temperatures calculated from changes in CO_2_ radiative forcing with *S* = 1.35 °C/(W/m^2^) added to the pre-industrial global surface land temperature *T*_s_ = 8 °C (blue), and (**B**,**C**,**D**) their frequency distributions. (**E**) Historical (red)^11^ and *Glaciator* (blue) changes in atmospheric concentration of CO_2_, and (**F**,**G**) their frequency distributions.

The *Glaciator* can be regarded as a case of slow passage through a Hopf bifurcation^51,52^. Parameters *α* and *β* change slowly with respect to time and, as exhibited in the simulations, solutions stay near the unstable stationary state after the point (*α*, *β*) has crossed the curve of instantaneous Hopf bifurcation; oscillations emerge only after a delay. As already mentioned, Hopf bifurcation is crossed ca. 5 Myr ago, but large oscillations appear ca. 2 Myr later (Fig. 7). These slow passages through bifurcations have been observed in other climate models^6,53^.

### Periodicities of past climate changes

The *Glaciator* has two adjustable parameters (*β*_0_ and *ω*) and one parameter with constrained values (0.84 < * α*(*t*) < 0.925) over the last 5 Myr. The values of three parameters are imposed by their assigned physical meanings: *ε* ≈ 33*ω*, *σ* ≈ 3.3*ρ* and *ρ* = *k*_7_/(*k*_1_*C*) = 0.11. The characteristic concentration allows for the conversion between the adimensional variables *x* and *y* to H_2_O and CO_2_ pressures. Using the parameters in Table 1, the *Glaciator* gives *x* = 95.5 at *t* = 0 in the absence of a CO_2_ pulse (i.e. *p*_H2O_ = 11.6 matm), and* y* = 2.19 (i.e., *p*_CO2_ = 262 ppm) at *t* = 0. These absolute values are in reasonable agreement with the pre-industrial CO_2_ pressure of 280 ppm and with the water vapor pressure expected for the temperature of pre-industrial times discussed in “Methods” section. The calculated atmospheric CO_2_ in the Last Glacial Maximum (LGM), *p*_CO2_ = 171 ppm, is in good agreement with the Antarctic ice-core record of 185 ppm^54^. These results come from the assignment of the timescales and not from an arbitrary scaling.

Figure 7 compares reconstructions of geological records with simulations of CO_2_ concentration and temperature over the last 5 Myr using the parameters in Table 1. The absolute values of CO_2_ pressure were obtained using the characteristic concentration. The temperature was estimated adding changes in CO_2_ radiative forcing to the pre-industrial global surface land temperature *T*_s_ = 8°C^55^.

The average global surface temperature change (∆*T*) is often related to radiative forcing changes (∆*R*) externally imposed at the top of the atmosphere by the long-wavelength absorption of greenhouse gases. The most important of them is CO_2_, and its concentration changes have been associated with global temperature changes over the last 420 million years^56^. Temperature and radiative forcing are related by the climate sensitivity parameter *S*

9 $$\Delta T = S\Delta R$$

The change in radiative forcing when the concentration of CO_2_ changes from that of a reference state (*C*_0_) to the state studied (*C*) is given by^40^

10 $$\Delta R = {5}.{\text{35 ln }}(C/C_{0} )$$

where the constant is in units of W/m^2^. Studies of past temperature changes showed that changing CO_2_ concentrations by a factor of 2 is consistent with ∆*T* = 2.8 °C, which is within the range 2.3–3.0 °C suggested by various climate models^56^. Taking ∆*T* = 2.8 °C for ∆*R* = 3.7 W/m^2^, gives *S* = 0.76 °C/(W/m^2^). An alternative approach for the long timescales considered here is to include slow feedbacks in the CO_2_ sensitivity parameter and employ a higher value, *S* = 1.35 °C/(W/m^2^)^1^. We adopted this approach for temperature calculations of the past.

The Mg/Ca ratios of foraminifera are one of the most reliable proxies of the paleo-temperatures of seawater^9,57^. A simple relation to obtain deep-sea temperature (in °C) was proposed^57^

11 $${{{\text{Mg}}} \mathord{\left/ {\vphantom {{{\text{Mg}}} {{\text{Ca}}}}} \right. \kern-\nulldelimiterspace} {{\text{Ca}}}} = 1 + 0.1T_{w}$$

Figure 7 uses this relation and the Mg/Ca ratios available from a 1.5-million-year record^9^ to obtain the corresponding Antarctic deep-sea temperatures. The benthic δ^18^O records of the LR stack^7^ were aligned with *T*_w_ from the Mg/Ca ratios to extend the estimated paleo-temperatures to 5 Myr ago.

**Figure 8:**
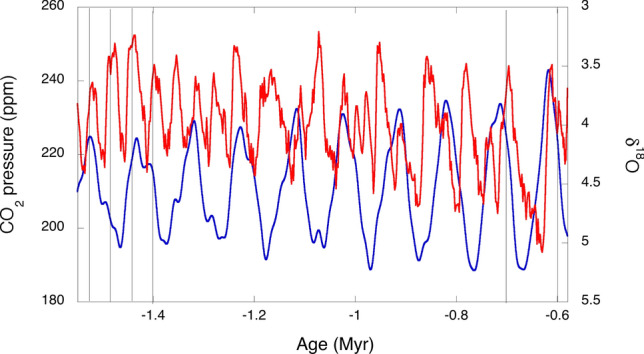
Mid-Pleistocene Transition. The benthic δ^18^O records of the LR04 stack (red)^7^ are compared with the changes in CO_2_ calculated with the *Glaciator* (blue). The vertical lines are separated by 42 kyr on the left and by 100 kyr on the right.

Calculated global surface temperatures (*T*_s_) are 3 to 5 °C higher than historical Antarctic deep-sea temperatures (*T*_w_), as expected. Remarkably, 5 Myr ago both exhibited a dominant period of 42 kyr, which changed to a dominant 100 kyr period ca. 1 Myr ago. This reflects the change in the main driver of climate from orbital forcing due to obliquity to a limit-cycle oscillation. Figure 8 presents a detail of the MPT. Ca. 2 Myr ago a stronger 100 kyr period started imposing on the older 42 kyr period to become clearly dominant in the last 1 Myr. According to the *Glaciator*, this change results from the increase of *α*(*t*) in the Pleistocene with the expansion of C4 plants, which increased the efficiency of photosynthesis and led to the crossing of a Hopf bifurcation.

The relative changes in terrestrial carbon stock can also be compared with observations. The extreme values of *z* in the last cycle of the *Glaciator* are 216 and 278, i.e., CH_2_O changes by ca. 24%. A recent estimate of the total carbon stocks (soils and vegetation) is 2,807 PgC^58^. Data-based estimates of the difference between the LGM and pre-industrial land carbon storage range from 330 PgC^59^ to 821 PgC^60^ less carbon in the LGM, which correspond to deficits between 10 and 30%. Hence, the change in terrestrial carbon stock given by the oscillator is consistent with the current estimates.

### Climate response to a CO_2_ pulse

**Figure 9:**
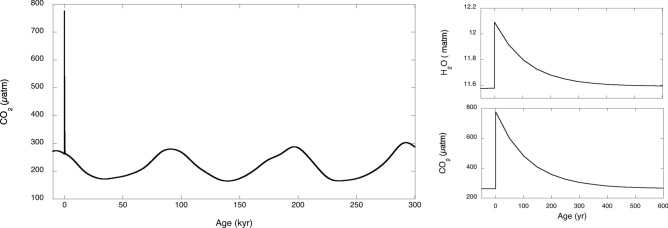
Predicted changes in the CO_2_ and water vapor after a CO_2_ pulse of 1,000 PgC.

Industrialization released approximately 300 PgC and business-as-usual predictions indicate a total release of 1,000 PgC (471 ppm of CO_2_) by the end of the century^42^. Climate models are often asked to predict the relaxation time of such a pulse on the current *p*_CO2_ level. In the *Glaciator*, the CO_2_ pulse comes with a H_2_O pulse. The scenario in Fig. 9 is a simulation of an instantaneous pulse of 1,000 PgC at present time, with the corresponding increase in water vapor, followed by relaxation assuming that all other parameters remain constant. Figure 8 shows that the relaxation time is approximately 100 years but the decay is non-exponential. Half of the CO_2_ pulse to the atmosphere is removed in 85 years. The IPCC estimate is that, for an emission pulse of about 1,000 PgC, about half is removed within a few decades^40^. Using the climate sensitivity parameter more appropriate for short timescales, *S* = 0.76 °C/(W/m^2^), this CO_2_ pulse corresponds to ∆*T* = 4.4 °C.

Figure 9 also illustrates a major difference between the *Glaciator* and simulations that do not take into account the dominant 100-kyr period of climate change. The CO_2_ pulse will eventually be absorbed and a new glacial cycle will begin. The next glacial maximum is predicted to occur in 50 kyr and be cooler than the preceding ones. This is due to the approach to the limit cycle shown in Fig. 4. The amplitude of the cycles depends on the final value of *α*, *α*(*∞*). Historical records show that the mid Pleistocene transition from 40-kyr to 100-kyr dominant cycles took more than half a million years. We estimate that the approach to the limit cycle started 5 Myr ago when *α*(*t*) became larger than 0.84 for *β*_0_ = 0.46, and the Hopf bifurcation was first crossed. However, as discussed in “Methods” section, the attraction to the limit cycle just after the bifurcation is very weak.

## Discussion

Our work addresses the internal Earth system mechanism that drives climate changes. We propose that this mechanism is an oscillator with the following steps: (i) photosynthesis uses CO_2_ and H_2_O to generate high-energy intermediates that store free energy for an extended period of time; (ii) the stored energy is released at the same time as CO_2_ and H_2_O that re-initialize the cycle; (iii) the minimal sufficient conditions for oscillatory behavior are met due to the presence of an autocatalytic step. According to this mechanism, the expansion of C4 plants, with the concomitant increase in radiation use efficiency of photosynthesis, triggered glaciation cycles.

The *Glaciator* with meaningful timescales leads to intrinsic oscillations consistent with the periods observed in records of historical climate changes, and to a good agreement with the absolute values of CO_2_ and H_2_O in the atmosphere over the last 5 Myr. This simple conceptual model explains major puzzles of past climate dynamics: the absence of a strong 20 kyr precession signal^8^ (Fig. 7F), the MPT of 41 kyr cycles to 100 kyr cycles^61^ (Figs. 7C, F), the onset of glaciation cycles one million years ago with a dominant 100-kyr frequency^22^ (Fig. 8), and the Holocene temperature conundrum^62^ (Fig. 7D).

An unexpected prediction of the *Glaciator* is that further expansion of C4 plants will drive more pronounced glaciations in the future. Climate is a complex dynamic system and the simple conceptual model presented here only captures the most fundamental mechanisms underlying its dynamics. Nevertheless, it seems that extreme climate changes may happen in the next 50 kyr. Feedback mechanisms not included in the *Glaciator* may change this view, but our present understanding suggests that dampening the amplitudes of climate changes may be achieved recovering land from C4 plants to C3 plants.

The *Glaciator* is open to improvements as our knowledge of the various processes responsible for atmospheric CO_2_ release and removal evolves. Regardless of possible refinements, in their present forms our oscillators reveal that closed photochemical systems are capable of producing sustained oscillations of their chemical compositions when absorbing light from a constant irradiation source.

## Methods

### Numerical simulations

Simulations were carried with Matlab R2018a. We used the ode45 solver to integrate each of the systems of differential equations: the *Coimbrator* and the *Glaciator*. This solver is based in a Runge–Kutta–Fehlberg method. In both cases we have fixed a relative error tolerance of 10^–12^.

The *Coimbrator* is a closed system of autonomous differential equations with a Hopf bifurcation. The Hopf bifurcation is supercritical and oscillations arise for parameter values below the bifurcation curve. The *Glaciator* is described by non-autonomous differential equations because the parameters *α* and *β* are time-dependent: *α*(*t*) = *P*_C3_(*t*)*α*_PC3_ + *P*_C4_(*t*)*α*_PC4_ and *β*(*t*) = *β*_0_[1 + 0.09*F*(*t*)]. Note that the expression for *β*(*t*) involves an unknown function *F*(*t*) related with the summer insolation in the Northern Hemisphere available in the literature^17^. Values of *F*(*t*) are available in steps of 1 kyr along the last 5 Myr. However, a sample of values of *F* is not enough for numerical simulations. We approach the values of *F*(*t*) at arbitrary *t* with two different methods. On one hand, when running the *Glaciator* to get simulations of the last 5 Myr we use a linear interpolation in each time interval corresponding to consecutive sample points. Alternatively, we have used the same sample points to get a Fourier series approaching *F*(*t*) in the whole period of 5 Myr. The good fitting obtained is illustrated in Fig. 1A. Both methods are used to obtain the simulations for the past, but no remarkable sensitivity is observed. To get extrapolations of the insolation values in the future, we have used the approach of the Fourier series. In the case of the *Glaciator*, when running the simulation in the past, initial time is fixed in the value − 5 Myr and the numerical integration extends up to present time. Initial conditions for the dependent variables are chosen close to the equilibrium point of the system when *α*(*t*) = *P*_C3_(*t*)*α*_PC3_ + *P*_C4_(*t*)*α*_PC4_ and *β* = *β*_0_, namely, *x* = 133.8721, *y* = 1.710171, *z* = 245.4826.

### Reduction of variables in the *Coimbrator*

The *Coimbrator* is defined by the reactions

12 $$\begin{aligned} & {\text{A}}\mathop{\longrightarrow}\limits^{{h\nu_{1} }}{\text{C}} \\ & {\text{C}} + {\text{Y}}\mathop{\longrightarrow}\limits^{{k_{1} }}{\text{B}} \\ & {\text{X}} + {\text{Y}}\mathop{\longrightarrow}\limits^{{k_{2} }}{\text{A}} \\ & {\text{B}}\mathop{\longrightarrow}\limits^{{h\nu_{2} }}{\text{D}} \\ & {\text{D}} + {\text{X}}\mathop{\longrightarrow}\limits^{{k_{3} }}{\text{Z}} + 2{\text{X}} \\ & {\text{Z}}\mathop{\longrightarrow}\limits^{{k_{4} }}2{\text{Y}} \\ \end{aligned}$$

The time dependence of all the chemical species involved in the model is described by

13 $$\begin{aligned} & \frac{{d\left[ {\text{A}} \right]}}{dt} = - h_{1} \left[ {\text{A}} \right] + k_{2} \left[ {\text{X}} \right]\left[ {\text{Y}} \right] \, \quad \quad \frac{{d\left[ {\text{C}} \right]}}{dt} = h_{1} \left[ {\text{A}} \right] - k_{1} \left[ {\text{C}} \right]\left[ {\text{Y}} \right] \\ & \frac{{d\left[ {\text{B}} \right]}}{dt} = k_{1} \left[ {\text{C}} \right]\left[ {\text{Y}} \right] - h_{2} \left[ {\text{B}} \right] \, \quad \quad \quad \frac{{d\left[ {\text{D}} \right]}}{dt} = h_{2} \left[ {\text{B}} \right] - k_{3} \left[ {\text{D}} \right]\left[ {\text{X}} \right] \\ & \frac{{d\left[ {\text{X}} \right]}}{dt} = k_{3} \left[ {\text{D}} \right]\left[ {\text{X}} \right] - k_{2} \left[ {\text{X}} \right]\left[ {\text{Y}} \right] \\ & \frac{{d\left[ {\text{Y}} \right]}}{dt} = - k_{1} \left[ {\text{C}} \right]\left[ {\text{Y}} \right] - k_{2} \left[ {\text{X}} \right]\left[ {\text{Y}} \right] + 2k_{4} \left[ {\text{Z}} \right] \\ & \frac{{d\left[ {\text{Z}} \right]}}{dt} = k_{3} \left[ {\text{D}} \right]\left[ {\text{X}} \right] - k_{4} \left[ {\text{Z}} \right] \\ \end{aligned}$$

where the photon fluxes were represented by *h*_1_ and *h*_2_. If A, B, C and D represent species in the solid state, by definition they have unitary concentrations. Hence, [A], [B], [C] and [D] are constant, and the system above can be simplified to the following set of differential equations

14 $$\begin{aligned} & \frac{{d\left[ {\text{X}} \right]}}{dt} = k_{3} \left[ {\text{D}} \right]\left[ {\text{X}} \right] - k_{2} \left[ {\text{X}} \right]\left[ {\text{Y}} \right] \\ & \frac{{d\left[ {\text{Y}} \right]}}{dt} = - k_{1} \left[ {\text{C}} \right]\left[ {\text{Y}} \right] - k_{2} \left[ {\text{X}} \right]\left[ {\text{Y}} \right] + 2k_{4} \left[ {\text{Z}} \right] \\ & \frac{{d\left[ {\text{Z}} \right]}}{dt} = k_{3} \left[ {\text{D}} \right]\left[ {\text{X}} \right] - k_{4} \left[ {\text{Z}} \right] \\ \end{aligned}$$

It is convenient to express these equations in terms of dimensionless variables to obtain a general solution. We follow the procedure of Field and Noyes^32^ to cast the concentrations of X, Y and Z in the dimensionless variables *x*, *y*, *z*. We define *g* = *k*_1_*C* as a pseudo-first order rate under continuous irradiation if *A* is not depleted from the system. Thus, the quotient *g*/*k*_2_ has the dimensions of a concentration and is defined as the characteristic concentration. This is used to make the following dimensionless measures of the species:

15 $$x = \frac{{k_{2} }}{g}\left[ {\text{X}} \right] \,\quad y = \frac{{k_{2} }}{g}\left[ {\text{Y}} \right] \,\quad z = \frac{{k_{2} }}{g}\left[ {\text{Z}} \right] \, \tau = gt$$

The characteristic concentration *g*/*k*_2_ can later be used to obtain the values of X, Y and Z in units of concentration. The same procedure is applied to the conversion of time *t* in dimensionless variable *τ*. The derivatives become

16 $$\begin{aligned}&\frac{{d\left[ {\text{X}} \right]}}{dt} = \frac{{{g \mathord{\left/ {\vphantom {g {k_{2} }}} \right. \kern-\nulldelimiterspace} {k_{2} }}}}{{{1 \mathord{\left/ {\vphantom {1 g}} \right. \kern-\nulldelimiterspace} g}}}\frac{dx}{{d\tau }} = \frac{{g^{2} }}{{k_{2} }}\frac{dx}{{d\tau }} \,\\ &\frac{{d\left[ {\text{Y}} \right]}}{dt} = \frac{{{g \mathord{\left/ {\vphantom {g {k_{2} }}} \right. \kern-\nulldelimiterspace} {k_{2} }}}}{{{1 \mathord{\left/ {\vphantom {1 g}} \right. \kern-\nulldelimiterspace} g}}}\frac{dy}{{d\tau }} = \frac{{g^{2} }}{{k_{2} }}\frac{dy}{{d\tau }} \,\\ &\frac{{d\left[ {\text{Z}} \right]}}{dt} = \frac{{{g \mathord{\left/ {\vphantom {g {k_{2} }}} \right. \kern-\nulldelimiterspace} {k_{2} }}}}{{{1 \mathord{\left/ {\vphantom {1 g}} \right. \kern-\nulldelimiterspace} g}}}\frac{dz}{{d\tau }} = \frac{{g^{2} }}{{k_{2} }}\frac{dz}{{d\tau }}\end{aligned}$$

Making the appropriate replacements we obtain a simplified system of differential equations, in dimensionless units

17 $$\begin{aligned} & \frac{{{\text{d}}x}}{{{\text{d}}\tau }} = \alpha x - xy \\ & \frac{{{\text{d}}y}}{{{\text{d}}\tau }} = - y - xy + 2\beta z \\ & \frac{{{\text{d}}z}}{{{\text{d}}\tau }} = \alpha x - \beta z \\ \end{aligned}$$

where *α* = (*k*_3_*D*)/(*k*_1_*C*) and *β* = *k*_4_/(*k*_1_*C*).

### Hopf bifurcation in the *Coimbrator*

In a physically meaningful system all the concentrations [C], [D], [X], [Y] and [Z] and all the constants *k*_1_, *k*_2_, *k*_3_ and *k*_4_ are positive. This three-dimensional system has two equilibrium points

18 $$O = \left( {0,0,0} \right) \,\quad P = \left( {1,\alpha ,\frac{\alpha }{\beta }} \right)$$

The Jacobian matrix of the vector field is

19 $$J\left( {x,y,z} \right) = \left[ {\begin{array}{*{20}c} {\alpha - y} & { - x} & 0 \\ { - y} & { - 1 - x} & {2\beta } \\ \alpha & 0 & { - \beta } \\ \end{array} } \right]$$

The characteristic polynomial at *O* is

20 $$p_{O} \left( \lambda \right) = det\left( {\left[ {\begin{array}{*{20}c} {\alpha - \lambda } & 0 & 0 \\ 0 & { - 1 - \lambda } & {2\beta } \\ \alpha & 0 & { - \beta - \lambda } \\ \end{array} } \right]} \right)$$

and gives the characteristic equation at *O*

21 $$\lambda^{3} + \left( {1 - \alpha + \beta } \right)\lambda^{2} + \left( { - \alpha + \beta - \alpha \beta } \right)\lambda - \alpha \beta = 0$$

The eigenvalues of *J*(0,0,0) are *λ*_1_ = *α*, *λ*_2_ = – 1, and *λ*_3_ = – *β*. Hence *O* is an equilibrium point of saddle type. Moreover, one can check that *x* = 0 is an invariant plane, namely, the stable invariant manifold at *O.* The unstable invariant manifold is tangent to *y* = *z* = 0.

The characteristic polynomial at *P* is

22 $$p_{P} \left( \lambda \right) = det\left( {\left[ {\begin{array}{*{20}c} {0 - \lambda } & { - 1} & 0 \\ { - \alpha } & { - 2 - \lambda } & {2\beta } \\ \alpha & 0 & { - \beta - \lambda } \\ \end{array} } \right]} \right)$$

and gives the characteristic equation at *P*

23 $$\lambda^{3} + \left( {\beta + 2} \right)\lambda^{2} + \left( {2\beta - \alpha } \right)\lambda + \alpha \beta = 0.$$

There are pure imaginary eigenvalues if and only if

24 $$\alpha \beta = \left( {\beta + 2} \right)\left( {2\beta - \alpha } \right) \Leftrightarrow \alpha = \frac{\beta (\beta + 2)}{{\beta + 1}}$$

and

25 $$2\beta - \alpha > 0.$$

Condition (25) is satisfied along the curve defined by (24) for all *β* > 0. These eigenvalues are $\lambda_{ \pm } = \pm i\sqrt {2\beta - \alpha }$. The third eigenvalue *λ*_3_ = –(*β* + 2) is negative for all *β* > 0. The distinction between subcritical and supercritical Hopf bifurcation depends on the value of the first Lyapunov coefficient^36^. Although such coefficient can be obtained for the given system, calculations are quite laborious and we prefer to use MATCONT^63^, a MATLAB numerical continuation package for bifurcation analysis, to check that the Hopf bifurcation is supercritical (see Fig. 3).

### Reduction of variables in the *Glaciator*

The *Glaciator* is defined by the reactions

26 $$\begin{aligned} & {\text{A}}\mathop{\longrightarrow}\limits^{{h\nu_{1} }}{\text{C}} \\ & {\text{C}} + {\text{Y}}\mathop{\longrightarrow}\limits^{{k_{1} }}{\text{B}} \\ & {\text{X}} + {\text{Y}}\mathop{\longrightarrow}\limits^{{k_{2} }}{\text{A}} \\ & {\text{B}}\mathop{\longrightarrow}\limits^{{h\nu_{2} }}{\text{D}} \\ & {\text{D}} + {\text{X}}\mathop{\longrightarrow}\limits^{{k_{3} }}{\text{Z}} + 2{\text{X}} \\ & {\text{Z}}\mathop{\longrightarrow}\limits^{{k_{4} }}2{\text{Y}} \\ & {\text{X}}\mathop{\longrightarrow}\limits^{{k_{5} }}{\text{X}}_{0} \\ & {\text{X}}_{0} \mathop{\longrightarrow}\limits^{{k_{6} }}{\text{X}} \\ & {\text{Y}}\mathop{\longrightarrow}\limits^{{k_{7} }}{\text{Y}}_{0} \\ & {\text{Y}}_{0} \mathop{\longrightarrow}\limits^{{k_{8} }}{\text{Y}} \\ \end{aligned}$$

The time dependence of all the chemical species involved in the *Glaciator* is described by

27 $$\begin{gathered} \frac{{d\left[ {\text{A}} \right]}}{dt} = - h_{1} \left[ {\text{A}} \right] + k_{2} \left[ {\text{X}} \right]\left[ {\text{Y}} \right] \,\quad \frac{{d\left[ {\text{C}} \right]}}{dt} = h_{1} \left[ {\text{A}} \right] - k_{1} \left[ {\text{C}} \right]\left[ {\text{Y}} \right] \hfill \\ \frac{{d\left[ {\text{B}} \right]}}{dt} = k_{1} \left[ {\text{C}} \right]\left[ {\text{Y}} \right] - h_{2} \left[ {\text{B}} \right] \,\quad \frac{{d\left[ {\text{D}} \right]}}{dt} = h_{2} \left[ {\text{B}} \right] - k_{3} \left[ {\text{D}} \right]\left[ {\text{X}} \right] \hfill \\ \frac{{d\left[ {\text{X}} \right]}}{dt} = - k_{2} \left[ {\text{X}} \right]\left[ {\text{Y}} \right] + k_{3} \left[ {\text{D}} \right]\left[ {\text{X}} \right] - k_{5} \left[ {\text{X}} \right] + k_{6} \left[ {{\text{X}}_{0} } \right] \hfill \\ \frac{{d\left[ {\text{Y}} \right]}}{dt} = - k_{1} \left[ {\text{C}} \right]\left[ {\text{Y}} \right] - k_{2} \left[ {\text{X}} \right]\left[ {\text{Y}} \right] + 2k_{4} \left[ {\text{Z}} \right] - k_{7} \left[ {\text{Y}} \right] + k_{8} \left[ {{\text{Y}}_{0} } \right] \hfill \\ \frac{{d\left[ {\text{Z}} \right]}}{dt} = k_{3} \left[ {\text{D}} \right]\left[ {\text{X}} \right] - k_{4} \left[ {\text{Z}} \right] \hfill \\ \end{gathered}$$

For constant [A], [B], [C] and [D], this reduces to following set of differential equations

28 $$\begin{aligned} & \frac{{d\left[ {\text{X}} \right]}}{dt} = - k_{2} \left[ {\text{X}} \right]\left[ {\text{Y}} \right] + k_{3} \left[ {\text{D}} \right]\left[ {\text{X}} \right] - k_{5} \left[ {\text{X}} \right] + k_{6} \left[ {{\text{X}}_{0} } \right] \\ & \frac{{d\left[ {\text{Y}} \right]}}{dt} = - k_{1} \left[ {\text{C}} \right]\left[ {\text{Y}} \right] - k_{2} \left[ {\text{X}} \right]\left[ {\text{Y}} \right] + 2k_{4} \left[ {\text{Z}} \right] - k_{7} \left[ {\text{Y}} \right] + k_{8} \left[ {{\text{Y}}_{0} } \right] \\ & \frac{{d\left[ {\text{Z}} \right]}}{dt} = k_{3} \left[ {\text{D}} \right]\left[ {\text{X}} \right] - k_{4} \left[ {\text{Z}} \right] \\ \end{aligned}$$

The following meanings can be assigned to terms in the equations above: *k*_2_[X][Y]—land uptake of H_2_O and CO_2_, *k*_3_[D][X]—a step in the biosynthesis of CH_2_O (e.g., condensation reactions in the formation of a glycosidic bond to make cellulose or in the formation of a peptide bond to yield a protein), *k*_5_[X]—condensation of water, *k*_6_[X_0_]—evaporation of water, *k*_4_[Z]—combustion/respiration, *k*_7_[Y]—loss of CO_2_ into the ocean leading to silicate weathering, *k*_7_[Y]—release of CO_2_ from the ocean in all its forms. As mentioned above and further discussed below, 1/(*k*_1_*C*) is the characteristic time and is associated with more than one physical process in this simplified description of the Earth system.

Making, as for the *Coimbrator*, *g* = *k*_1_*C*, the quotient *g*/*k*_2_ is defined as the characteristic concentration. Additionally, 1/*g* is defined as the characteristic time. The characteristic time and concentration are used to obtain the dimensionless variables

29 $$x = \frac{{k_{2} }}{g}\left[ {\text{X}} \right]\quad x_{0} = \frac{{k_{2} }}{g}\left[ {{\text{X}}_{0} } \right]\quad y = \frac{{k_{2} }}{g}\left[ {\text{Y}} \right]\quad y_{0} = \frac{{k_{2} }}{g}\left[ {\text{Y}} \right]\quad z = \frac{{k_{2} }}{g}\left[ {\text{Z}} \right]\quad \tau = gt$$

In terms of the adimensional variables, we obtain

30 $$\begin{aligned} & \frac{{{\text{d}}x}}{{{\text{d}}\tau }} = \varepsilon + \left( {\alpha - \sigma } \right)x - xy \\ & \frac{{{\text{d}}y}}{{{\text{d}}\tau }} = \omega - \left( {1 + \rho } \right)y - xy + 2\beta z \\ & \frac{{{\text{d}}z}}{{{\text{d}}\tau }} = \alpha x - \beta z \\ \end{aligned}$$

where

31 $$\begin{aligned} & \alpha = \frac{{k_{3} \left[ {\text{D}} \right]}}{{k_{1} \left[ {\text{C}} \right]}} \,\quad\quad \sigma = \frac{{k_{5} }}{{k_{1} \left[ {\text{C}} \right]}} \,\quad\quad \varepsilon { = }\frac{{k_{6} x_{0} }}{{k_{1} \left[ {\text{C}} \right]}} \\ & \beta = \frac{{k_{4} }}{{k_{1} \left[ {\text{C}} \right]}} \,\quad\quad \rho = \frac{{k_{7} }}{{k_{1} \left[ {\text{C}} \right]}} \,\quad\quad \omega { = }\frac{{k_{8} y_{0} }}{{k_{1} \left[ {\text{C}} \right]}} \\ \end{aligned}$$

Considering that *k*_6_*x*_0_ and *k*_8_*y*_0_ are proportional to the water evaporation and to the CO_2_ desorption fluxes, respectively, the following relation must be obeyed

32 $$\frac{{k_{6} x_{0} }}{{k_{8} y_{0} }} = \frac{\varepsilon }{\omega } = \frac{{{\text{water}}\,{\text{evaporation}}\,{\text{flux}}}}{{{\text{CO}}_{2} \,{\text{desorption}}\,{\text{flux}}}}$$

The desorption flux of CO_2_ from water at 313 K is *j*_CO2_ ≈ 7 × 10^–4^ mol/(m^2^ s)^64^. At this temperature, the water evaporation flux from seawater ranges from 1.5 × 10^–2^ to 4.6 mol/(m^2^ s), depending on the air velocity^65^. Using *j*_H2O_ ≈ 2.3 × 10^–2^ mol/(m^2^ s) we estimate *ε* ≈ 33*ω*.

### Constrains of the parameters in the *Glaciator*

The saturation vapor pressure can be calculated from an approximation to the Clausius–Clapeyron equation

33 $$e_{0}^{*} = 17.044exp\left[ {a\left( {T - 288} \right)} \right]$$

where the constants where chosen to yield the vapor pressure in mbar, *T* is in K and *a* = 0.064 K^–1^ ^66^. Using the average Earth surface land temperature *T*_s_ = 8 °C to represent pre-industrial global temperature (ca. 1900)^55^, we obtain *e*_0_* ≈ 10.9 mbar, which corresponds to *p*_H2O_ ≈ 11 matm.

Water vapor equilibrates relatively rapidly with liquid water (*k*_5_*x* ≈ *k*_6_*x*_0_). Assuming CO_2_ in the atmosphere also equilibrates relatively rapidly with CO_2_ dissolved in the sea (*k*_7_*y* ≈ *k*_8_*y*_0_), the following relations can be established

34 $$\frac{\sigma }{\rho } = \frac{{k_{5} }}{{k_{7} }} = \frac{{k_{6} x_{0} }}{{k_{8} y_{0} }}\frac{y}{x} = \frac{\varepsilon }{\omega }\frac{y}{x}$$

Given that

35 $$\frac{{p_{{{\text{CO2}}}} }}{{p_{{{\text{H2O}}}} }} = \frac{Y}{X} = \frac{y}{x}$$

we reduced the number of variables needed to apply the *Glaciator* making *x* ≈ 10*y* and using *ε* ≈ 33*ω*, to set *σ* ≈ 3.3*ρ*.

### Hopf bifurcation in the *Glaciator*

The *Glaciator*, as the *Coimbrator*, has two equilibrium points. We have to study the following system of nonlinear equations

36 $$\begin{aligned} & \varepsilon + \left( {\alpha - \sigma } \right)x - xy = 0 \\ & \omega - \left( {1 + \rho } \right)y - xy + 2\beta z = 0 \\ & \alpha x - \beta z = 0 \\ \end{aligned}$$

From the third equation we obtain

37 $$z = \frac{x}{\beta }$$

After substituting *z* in the second equation we get

38 $$y = \frac{{\omega - \varepsilon + \left( {\alpha + \sigma } \right)x}}{\rho + 1}$$

and finally, substituting *y* in the first equation, we obtain the following equation for *x*

39 $$\varepsilon \left( {\rho + 1} \right) + \left[ {\left( {\alpha - \sigma } \right)\left( {\rho + 1} \right) - \omega + \varepsilon } \right]x - \left( {\alpha + \sigma } \right)x^{2} = 0$$

Since [(*α – ρ*)(*ρ* + 1) – *ω* + *ε*)^2^ + 4*ε *(*ρ* + 1) (*α* + *σ*)] > 0, we always have two equilibria. In this case the analytical characterization of the Hopf bifurcation is plausible but more involved. Using again, as we did for the *Coimbrator*, the package MATCONT, we obtain the results depicted in Fig. 6, for which we have fixed the values

40 $$\omega = {5},\quad \rho = 0.{11},\quad \varepsilon = { 33}\omega ,\quad \sigma = { 3}.{3}\rho$$

**Figure 10:**
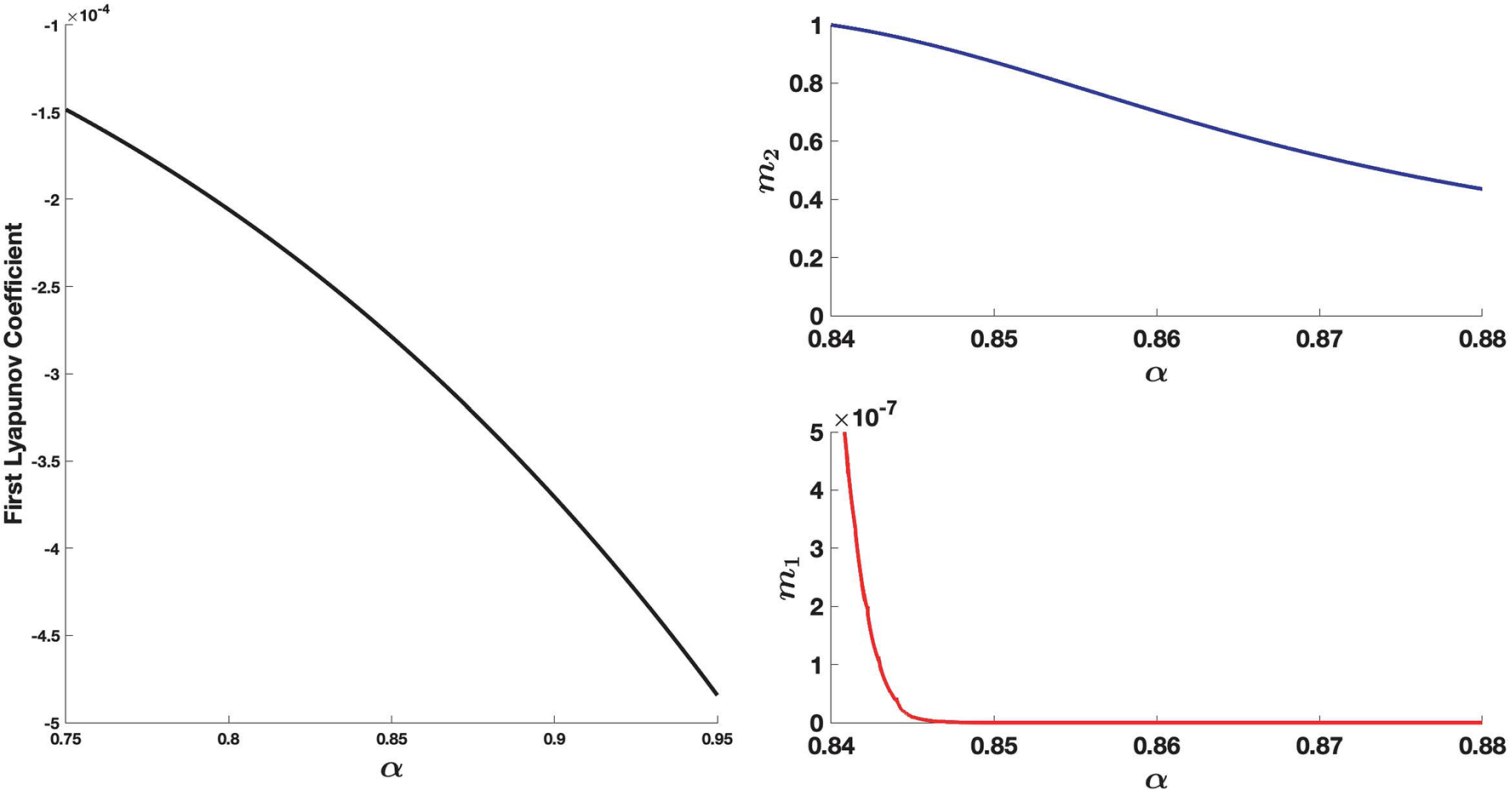
Left panel: Variation of the first Lyapunov coefficient along the Hopf bifurcation curve. Right panel: Variation of the Floquet multipliers associated to the periodic orbits emerging at *α* = 0.84 when *β* = 0.46.

As shown in Fig. 10, the first Lyapunov coefficient is negative in the whole range of parameters considered in Fig. 6 and hence the Hopf bifurcation is supercritical. A periodic orbit in a three-dimensional phase space has Floquet multipliers 1, *m*_1_ and *m*_2_ (*m*_1_ and *m*_2_ are the eigenvalues of the linearization of the first return map defined around the periodic orbit). If |*m*_i_|< 1, for *i* = 1,2, then the periodic orbit is attracting. The right panel in Fig. 10 shows the value of Floquet multipliers for the periodic orbits born at *α* = 0.84 when *β* = 0.46. Because the first Lyapunov coefficient is very small, the attraction of the limit cycle just after the bifurcation is very slow. For the computation of the first Lyapunov coefficient, instead of using the approximations obtained with MATCONT, we wrote our own code, designed for this specific model, to obtain more accurate numerical results.

### Populations of C3 and C4 plants

The value of *α* in Eq. (5) must take in consideration the population of C3 and C4 plants

41 $$\alpha \left( t \right) = P_{C3} \left( t \right)\alpha_{C3} + P_{C4} \left( t \right)\alpha_{C4}$$

**Figure 11:**
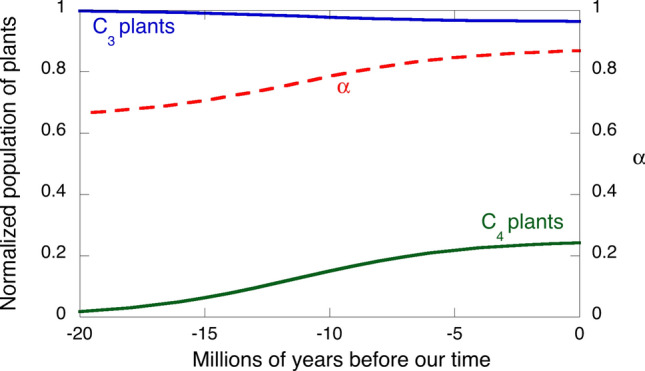
Left axis: changes in the relative populations of C3 and C4 plants, where the logistic equation was employed to express the explosive spread of C4 plants ca. 10 Myr ago at some cost of the C3 plants population. Right axis: value of *α* given the contributions of C3 and C4 plants weighted by their relative populations.

We simulate C4 plants expansion with the logistic equation often used to describe population dynamics

42 $$P_{C4} \left( t \right) = \frac{{N_{max} }}{{1 + \left( {\frac{{N_{max} - N_{0} }}{{N_{0} }}} \right)exp\left[ { - r\left( {t + t_{0} } \right)} \right]}}$$

where *N*_max_ is the population maximum at *t* = ∞, *N*_0_ is the population at – *t*_0_, which is selected to be the origin of the expansion, and *r* is the rate of population increase. The sudden C4 plants expansion occurred ca. 10 Myr ago^44^, which leads to *t*_0_ = 10 Myr. *N*_max_ = 0.25 was selected in view of the fact that C4 plants account for 25% of terrestrial photosynthesis. With these values of *t*_0_ and *N*_max_, the known profile of C4 plants expansion^44^ can be reproduced with the population *N*_0_ = 0.15 and the rate *r* = 0.3 Myr^–1^. The expansion of C4 plants was achieved, in part, at the expense of C3 plants. We modelled the reduction of C3 plants population as

43 $$P_{C3} \left( t \right) = 1 - 0.15P_{C4} \left( t \right)$$

where the factor 0.15 was selected to reflect evolution from nearly exclusive C3 vegetation 20 Myr ago to the composition of biomass today: 389.3 PgC of C3 vegetation and 18.9 PgC of C4 vegetation^67^. This corresponds to a fraction of C3 biomass of 0.954, whereas Eq. (43) gives *P*_C3_(0) = 0.96. Equation (43) assumes that only 15% of the increase in C4 plants is made at the cost of C3 plants. Figure 11 presents the changes in the populations of C3 and C4 plants. It also shows the changes in *α*(*t*) calculated with Eq. (41).

## Data Availability

Data described in this study will be available from the corresponding authors upon request.

## References

[1] Hönisch B, Hemming NG, Archer D, Siddall M, McManus JF (2009). Atmospheric carbon dioxide concentration across the mid-Pleistocene transition. Science.

[2] Huybers P (2011). Combined obliquity and precession pacing of late Pleistocene deglaciations. Nature.

[3] Crucifix M (2012). Oscillators and relaxation phenomena in Pleistocene climate theory. Philos. Trans. R. Soc. A.

[4] Martinez-Botí MA (2015). Plio-Pleistocene climate sensitivity evaluated using high-resolution CO_2_ records. Nature.

[5] Zeebe RE, Westerhold T, Littler K, Zachos JC (2017). Orbital forcing of the Paleocene and Eocene carbon cycle. Paleoceanography.

[6] Nyman KHM, Ditlevsen PD (2019). The middle Pleistocene transition by frequency locking and slow ramping of internal period. Clim. Dyn..

[7] Lisiecki LE, Raymo ME (2005). A Pliocene-Pleistocene stack of 57 globally distributed benthic d18O records. Paleoceanography.

[8] Patterson MO (2014). Orbital forcing of the East Antarctic ice sheet during the Pliocene and Early Pleistocene. Nature Geosci..

[9] Elderfield H (2012). Evolution of ocean temperature and ice volume through the mid-Pleistocene climate transition. Science.

[10] Erb MP, Broccoli AJ, Clement AC (2013). The contribution of radiative feedbacks to orbitally driven climate change. J. Climate.

[11] Petit JR (1999). Climate and atmospheric history of the past 420,000 years from the Vostok ice core, Anarctica. Nature.

[12] Uemura R (2018). Asynchrony between Antarctic temperature and CO_2_ associated with obliquity over the past 720,000 years. Nature Comm..

[13] Hays JD, Imbrie J, Shackleton NJ (1976). Variations in the Earth's Orbit: Pacemakes of the Ice Ages. Science.

[14] Jochum M (2012). True to Milankovitch: Glacial inception in the new community climate system model. J. Climate.

[15] Milankovitch, M. *Canon of Insolation and the Ice-Age Problem*. 848 (Israel Program for Scientific Translations, 1941).

[16] Berger A, Loutre MF (1991). Insolation values for the climate of the last 10 million years. Quat. Sci. Rev..

[17] Huybers P (2006). Early Pleistocene glacial cycles and the integrated summer insolation forcing. Science.

[18] Laskar J, Robutel P, Joutel F, Gastineau M, Correia ACM (2004). A long-term numerical solution for the insolation quantities of the Earth. Astron. Astrophys..

[19] Berger A, Loutre M-F, Yin Q (2010). Total irradiation during any time interval of the year using elliptic integrals. Quat. Sci. Rev..

[20] Raymo ME, Huybers P (2008). Unlocking the mysteries of the ice ages. Nature.

[21] Maslin MA, Brierley CM (2015). The role of orbital forcing in the Early Middle Pleistocene Transition. Quatern. Int..

[22] van Nes EH (2015). Causal feedbacks in climate changes. Nature Clim. Change.

[23] Daruka I, Ditlevsen PD (2016). A conceptual model for glacial cycles and the middle Pleistocene transition. Clim. Dyn..

[24] Huybers P (2007). Glacial variability over the last two million years: an extended depth-derived agemodel, continuous obliquity pacing, and the Pleistocene progression. Quat. Sci. Rev..

[25] Saltzman B, Maasch KA (1991). A first-order global model of late Cenozoic climate change. II. Further analysis based on a simplification of CO_2_ dynamics. Clim. Dyn..

[26] Lotka AJ (1920). Undamped oscillations derived from the law of mass action. J. Am. Chem. Soc..

[27] Volterra V (1926). Variazioni e fluttuazioni del numero d'individui in specie animali conviventi. Mem. Acad. Lincei Roma.

[28] Prigogine I, Lefever R (1968). Symmetry breaking instabilities in dissipative systems. II.. J. Chem. Phys..

[29] Prigogine I (1978). Time, structure, and fluctuations. Science.

[30] Drubi F, Ibáñez S, Rodriguez JA (2007). Coupling leads to chaos. J. Differ. Equ..

[31] Zaikin AN, Zhabotinsky AM (1970). Concentration wave propagation in two-dimensional liquid-phase self-oscillating system. Nature.

[32] Field RJ, Noyes RM (1974). Oscillations in chemical systems. IV. Limit cycle behavior in a model of a real chemical reaction. J. Chem. Phys..

[33] Noyes RM (1985). Effects of global constraints on permissible local behavior. Ber. Bunsenges. Phys. Chem..

[34] Epstein IR, Showalter K (1996). Nonlinear chemical dynamics: Oscillations, patterns, and chaos. J. Phys. Chem..

[35] Li R-S, Ross J (1991). Chemical instabilities in closed systems with illumination. J. Phys. Chem..

[36] Strogatz SH (2015). Nonlinear Dynamics and Chaos: With Applications to Physics, Biology, Chemistry, and Engineering.

[37] Sel'kov EE (1968). Self-oscillations in glycolysis 1. A simple kinetic model. Eur. J. Biochem..

[38] Wilhelm T, Heinrich R (1995). Smallest chemical reaction system with Hopf bifurcation. J. Math. Chem..

[39] Berner RA (2005). The Phanerozoic Carbon Cycle: CO_2_ and O_2_.

[40] IPCC. *Climate Change 2013: The Physical Science Basis. Contribution of Working Group I to the Fifth Assessment Report of the Intergovernmental Panel on Climate Change*. (Cambridge University Press, 2013).

[41] Eby M (2009). Lifetime of antropogenic climate change: Millennial time scales of potential CO_2_ and surface temperature perturbations. J. Climate.

[42] Archer D (2009). Atmospheric lifetime of fossil fuel carbon dioxide. Annu. Rev. Earth Planet. Sci..

[43] Marcott SA (2014). Centennial-scale changes in the global carbon cycle during the last deglaciation. Nature.

[44] Edwards EJ, Osborne CP, Strömberg CAE, Smith SA, Consortium CG (2010). The origins of C4 grasslands: Integrating evolutionary and ecosystem science. Science.

[45] Sage RF (2004). The evolution of C_4_ photosynthesis. New Phytol..

[46] Seemann JR, Badger MR, Berry JA (1984). Variations in the specific activity of ribulose-1,5-bisphophate carboxylase between species utilizing different photosynthetic pathways. Plant Physiol..

[47] Sage RF, Zhu X-G (2011). Exploiting the engine of C_4_ photosynthesis. J. Exp. Botany.

[48] Raymo ME, Nisancioglu K (2003). The 41 kyr world: Milankovitch’s other unsolved mystery. Paleoceanography.

[49] Bosmans JHC, Hilgen FJ, Lourens LJ (2015). Obliquity forcing of low-latitude climate. Clim. Past.

[50] Kuechler RR, Dupont LM, Shefuß E (2018). Hybrid insolation forcing of Pliocene monsoon dynamics in West Africa. Clim. Past.

[51] Baer SM, Erneux T, Rinzel J (1989). The slow passage through a Hopf bifurcation: delay, memory effects, and resonance. SIAM J. Appl. Math..

[52] Hayes MG, Kaper TJ, Szmolyan P, Wechselberger M (2016). Geometric desingularization of degenerate singularities in the presence of fast rotation: A new proof of known results for slow passage through Hopf bifurcations. Indag. Math..

[53] Ashwin P, Ditlevsen P (2015). The middle Pleistocene transition as a generic bifurcation on a slow manifold. Clim. Dyn..

[54] Monnin E (2001). Atmospheric CO_2_ concentrations of the Last Glacial Termination. Science.

[55] Rohde R (2013). A new estimate of the average Earth surface land temperature spanning 1753 to 2011. Geoinform. Geostat. Overv..

[56] Royer DL, Berner RA, Park J (2007). Climate sensitivity constrained by CO_2_ concentrations over the past 420 million years. Nature.

[57] Elderfield H (2010). A record of bottom water temperature and seawater ∂18O for the Southern Ocean over the apst 440 kyr based on Mg/Ca of benthic foraminiferal Uvegina spp. Quat. Sci. Rev..

[58] Carvalhais N (2014). Global covariation of carbon turnover times with climate in terrestrial ecosystems. Nature.

[59] Ciais P (2012). Large inert carbon pool in the terrestrial biosphere during the Last Glacial Maximum. Nat. Geosci..

[60] Kaplan JO, Prentice IC, Knorr W, Valdes PJ (2002). Modeling the dynamics of terrestrial carbon storage since the Last Glacial Maximum. Geophys. Res. Lett..

[61] Snyder CW (2016). Evolution of global temperature over the past two million years. Nature.

[62] Liu Z (2014). The Holocene temperature conundrum. Proc. Natl. Acad. Sc. USA.

[63] Dhooge A, Govaerts W, Kuznetsov YA, Meijer HGE, Sautois B (2008). New features of the software MatCont for bifurcation analysis of dynamical systems. MCMDS.

[64] Tunnat A, Behr P, Görner K (2014). Desorption kinetics of CO2 from water and aqueous amine solutions. Energy Procedia.

[65] El-Dessouky HT, Ettouney HM, Alatiqi IM, Al-Shamari MA (2002). Evaporatoin rates from fresh and saline water in moving air. Ind. Eng. Chem. Res..

[66] Stephens GL (1990). On the relationship between water vapor over the oceans and sea surface temperature. J. Clim..

[67] Still CJ, Berry JA, Collatz GJ, Defries RS (2003). Global distribution of C3 and C4 vegetation: Carbon cycle implications. Global Geobiochem. Cycles.

